# Model-based iterative reconstruction for reduction of radiation dose in abdominopelvic CT: comparison to adaptive statistical iterative reconstruction

**DOI:** 10.1186/2193-1801-2-209

**Published:** 2013-05-07

**Authors:** Koichiro Yasaka, Masaki Katsura, Masaaki Akahane, Jiro Sato, Izuru Matsuda, Kuni Ohtomo

**Affiliations:** Department of Radiology, Graduate School of Medicine, The University of Tokyo, Tokyo, Japan; Kanto Rosai Hospital, Kanagawa, Japan

**Keywords:** Model-based iterative reconstruction, Adaptive statistical iterative reconstruction, Filtered back projection, Streak artifact, Image noise

## Abstract

**Purpose:**

To evaluate dose reduction and image quality of abdominopelvic computed tomography (CT) reconstructed with model-based iterative reconstruction (MBIR) compared to adaptive statistical iterative reconstruction (ASIR).

**Materials and methods:**

In this prospective study, 85 patients underwent referential-, low-, and ultralow-dose unenhanced abdominopelvic CT. Images were reconstructed with ASIR for low-dose (L-ASIR) and ultralow-dose CT (UL-ASIR), and with MBIR for ultralow-dose CT (UL-MBIR). Image noise was measured in the abdominal aorta and iliopsoas muscle. Subjective image analyses and a lesion detection study (adrenal nodules) were conducted by two blinded radiologists. A reference standard was established by a consensus panel of two different radiologists using referential-dose CT reconstructed with filtered back projection.

**Results:**

Compared to low-dose CT, there was a 63% decrease in dose-length product with ultralow-dose CT. UL-MBIR had significantly lower image noise than L-ASIR and UL-ASIR (all p<0.01). UL-MBIR was significantly better for subjective image noise and streak artifacts than L-ASIR and UL-ASIR (all p<0.01). There were no significant differences between UL-MBIR and L-ASIR in diagnostic acceptability (p>0.65), or diagnostic performance for adrenal nodules (p>0.87).

**Conclusion:**

MBIR significantly improves image noise and streak artifacts compared to ASIR, and can achieve radiation dose reduction without severely compromising image quality.

## Introduction

The increase in the use of computed tomography (CT) has raised concerns about the potential increase in the incidence of radiation-induced carcinogenesis. The estimated annual effective dose (ED) from medical radiation exposure per individual in the United States (US) population has increased about six-fold over the past quarter century, from 0.53 mSv in 1980 to 3.0 mSv in 2006 (Mettler et al. 2008). CT is the largest source (ED of 1.47 mSv) of medical exposure in 2006, and abdominopelvic CT amounts to almost one half of the entire CT collective dose (Mettler et al. 2008). Therefore, dose reduction in abdominopelvic CT examination, without remarkable loss of diagnostic performance, plays an important role in reducing radiation dose delivered to patients.

Several strategies have been employed to enable dose reductions during CT acquisition, such as tube current modulation, reduced tube voltage, use of a higher pitch and noise reduction filters (Kalra et al. 2004; McCollough et al. 2012). Although there are clinical studies that have evaluated dose reduction for abdominopelvic CT (Iannaccone et al. 2003; Niemann et al. 2011), further dose reductions are hindered by increased image noise and degraded image quality, mainly as a result of limitations of the standard filtered back projection (FBP) reconstruction algorithm currently used on most CT systems.

The iterative reconstruction (IR) algorithm has recently received much attention in CT because it has many advantages compared with conventional FBP techniques. IR generates a set of synthesized projections by accurately modeling the data collection process in CT. The model incorporates statistical information from the CT system (including photon statistics and electronic acquisition noise), and details of the system optics (including the size of each detector cell, dimensions of the focal spot, and the shape and size of each image voxel), to yield lower image noise and higher spatial resolution compared to FBP. One of the first IR algorithms released for clinical use was the adaptive statistical iterative reconstruction (ASIR) algorithm (GE Healthcare, Waukesha, WI, USA). ASIR is a hybrid IR that involves blending with FBP images, and it models only the photons and electronic noise that primarily affect image noise. ASIR significantly reduces image noise compared to the FBP algorithm, provides dose-reduced clinical images with preserved diagnostic value, and has been widely used for dose reduction in many CT systems (Prakash et al. 2010; Sagara et al. 2010; Martinsen et al. 2012; Singh et al. 2010; Desai et al. 2012; Mitsumori et al. 2012).

The recently developed model-based iterative reconstruction (MBIR) technique is a pure IR technique that does not involve blending with FBP images (i.e. no reconstruction kernel), and is mathematically more complex and accurate than ASIR (Beister et al. 2012; Yu et al. 2011; Thibault et al. 2007). MBIR not only incorporates modeling of photon and noise statistics like ASIR, but it also involves modeling of system optics. Phantom experiments have shown that MBIR provides a significant reduction in image noise and streak artifacts and a significant improvement in spatial resolution (Mieville et al. 2013; Husarik et al. 2012). However, up to this point, clinical studies that have assessed its effect on radiation dose reduction in patients are limited (Singh et al. 2012; Yamada et al. 2012; Katsura et al. 2012).

The purpose of this study was thus to evaluate the effect of MBIR on radiation dose reduction and compare image quality and diagnostic performance with ASIR in abdominopelvic CT.

## Materials and methods

This prospective clinical study was approved by the Human Research Committee of our Institutional Review Board. Written informed consent was obtained from each patient.

### Patients

The Radiology Information System at a single tertiary care center was checked to identify patients scheduled for unenhanced standard-of-care clinical abdominopelvic CT examinations. Patients were scheduled to undergo CT without intravenous contrast, as instructed by their attending physicians for any reason (e.g. no clinical indication for using contrast, history of a previous adverse reaction to iodine contrast media, or impairment in renal function). Inclusion criteria for the present study were as follows: age ≥50 years, patient scheduled for non-emergent unenhanced standard-of-care CT examination of the abdomen and pelvis, ability to give written informed consent, and the ability to hold one’s breath and remain still for at least 10 s. Patients who were unable to provide written informed consent, follow verbal commands for breath-holding, or remain still for the duration of CT acquisition were excluded. Women who were pregnant or were trying to get pregnant were also excluded. Each potential subject was given a detailed informed consent form written in simple language about the objective, method and risks of study participation. The study procedure, which involved an acquisition of referential-dose CT (RDCT) followed by low-dose CT (LDCT) and ultralow-dose CT (ULDCT), was explained to the subjects. They were also informed that the sum of the total radiation exposure from RDCT, LDCT, and ULDCT acquisitions would not exceed the radiation dose for standard-of-care abdominopelvic CT at our institution. The risks associated with study participation, particularly the possible influence on diagnostic performance, in which the referential dose in the present study was expected to be slightly lower compared to radiation doses for standard-of-care CT, were explained to the subjects in simple language. Subjects were also informed that they would not receive any remuneration or benefit from their participation in the study.

Between October 2011 to December 2011, 98 consecutive eligible patients were identified. Seven patients declined to participate in the study, and 91 gave informed consent to participation in the study. None withdrew from the study after signing the consent form. Images from 6 patients were selected using a random number table, used for training purposes (to understand the evaluation system), and subsequently eliminated from the remaining analyses. As a result, 85 patients (57 men and 28 women, mean patient age 69.9 ± 9.0 years, mean body weight 61.2 ± 12.2 kg) were included in the final analysis. Indications for CT were as follows; evaluation of malignant tumors (n=52), urolithiasis (n=15), abdominal aortic aneurysm (n=11), stricture of ureter (n=2), renal artery aneurysm (n=1), splenic artery aneurysm (n=1), inguinal hernia (n=1), subcutaneous tunnel infection (n=1), and evaluation of lymph node (n=1).

### CT data acquisition

RDCT followed by LDCT and ULDCT were acquired with a 64-row multidetector CT (Discovery CT750 HD; GE Healthcare, Waukesha, WI, USA). All patients in the study were able to undergo abdominopelvic CT in the supine position with both arms elevated and with a single breath-hold for each acquisition. In this prospective clinical study, RDCT, LDCT, and ULDCT were acquired with minimal differences in data acquisition conditions (with the exception of radiation dose). For instance, to minimize the positional difference between the three acquisitions for each patient, the time between each scan was kept to a minimum (about 10 s or less). To avoid contrast enhancement bias owing to the delay in imaging from the start of the injection, only unenhanced CT images were included in this study. Scanning parameters other than tube current were held constant. The remaining scanning parameters were as follows: tube voltage of 120 kilovolt peak, helical scan acquisition mode, pitch of 1.375:1, gantry rotation time of 0.4 s, field of view of 360 mm (adjusted to patient size), and detector configuration of 64 × 0.625 mm. All images were reconstructed with 0.625 mm thick axial slices, and images with increased slice thickness of 2.5 mm (by averaging) were also used for interpretation as necessary. Images with coronal/sagittal reformats were not used for evaluation in this study, since the preliminary results of phantom experiments indicated that MBIR and ASIR behave differently in terms of image noise when reformatted into coronal and sagittal slices (unpublished data). The STANDARD kernel (a proprietary kernel of GE Healthcare) was used for image reconstruction for FBP and ASIR (there is no concept of kernel for MBIR).

### Radiation dose settings

RDCT, LDCT, and ULDCT protocols involved the use of automatic tube current modulation (Auto mA 3D, GE Healthcare, Waukesha, WI). The operator-selected noise index (NI) level modulates the tube current during gantry rotation to achieve a predicted average statistical noise level in the images of the reconstruction slice thickness specified. Some previous study reports that ASIR (Prakash et al. 2010; Sagara et al. 2010; Desai et al. 2012; Mitsumori et al. 2012) and MBIR (Mieville et al. 2013; Singh et al. 2012) enabled reduced dose level of 59 – 75% and 14 – 33% compared with that of FBP, respectively. Thus, we targeted that the ED of ULDCT would be about 35 – 40% of that of LDCT. The ED of RDCT was targeted to be 70 – 75% of that of standard-of-care CT so that the diagnostic performance in RDCT would not be substantially lower than that of standard-of-care CT in our hospital. Image noise is known to inversely proportional to square root of radiation dose. In our hospital noise index of 10.6 for 5 mm slice thickness was used. Therefore, in the present study, fixed noise indices (5 mm slice thickness) of 12.3, 24.6 and 40.6 were calculated for RDCT, LDCT, and ULDCT, respectively to meet the radiation dose levels described above.

### Image reconstruction

Images for RDCT were reconstructed with FBP (R-FBP) and used for establishing a reference standard. Images for LDCT were reconstructed with blending of 50% FBP and 50% ASIR image data (ASIR50). A blending factor of 50% was chosen based on the literature (Prakash et al. 2010; Singh et al. 2010; Singh et al. 2012) and the recommendations of the vendor. Images for ULDCT were reconstructed with ASIR50 and MBIR. Blending with FBP does not apply to MBIR, as this is a pure IR technique. Thus, three axial image datasets (L-ASIR, UL-ASIR and UL-MBIR) were generated for each patient (255 image sets from 85 patients) and used for image interpretation, after removing patient information to allow blinded evaluation.

### Objective image analysis

Objective measurements were performed for the image data sets of 85 patients (255 image sets) on a workstation (Centricity RA1000; GE Healthcare) by a radiologist (K.Y.) with 2 years of imaging experience (1 year of experience with ASIR and 0.5 years of experience with MBIR). Circular or ovoid regions of interest (ROI) (approximately 10 mm in diameter) were placed in the abdominal aorta at the level of renal artery and in right iliopsoas muscle at the level of the first anterior sacral foramen. CT number (Hounsfield units [HU]) and standard deviation (i.e. objective image noise) were recorded. To avoid partial volume effect, peripheral part of the structure was not included in the ROI. Calcification and apparent intraluminal hematoma (in the abdominal aorta) were also avoided. To evaluate the radiation dose, the estimated CT dose index volume (CTDIvol) and dose–length product (DLP) were recorded for each image data set following completion of the CT examination, according to the dose report. ED (described in mSv) was calculated by multiplying 0.015 (mSv-mGy^-1^-cm^-1^) with DLP (Shrimpton et al. 2006).

### Subjective image analysis

Two radiologists (J.S. and I.M. 13 and 6 years of experience as radiologists, respectively) independently assessed the image sets of 85 patients (255 image sets) using a commercial viewer (EV Insite; PSP Corporation, Tokyo, Japan). Both had 3 years of ASIR experience and 0.5 years of MBIR experience at the time of the present study. To assess intraobserver agreement, 8 patients (24 image sets) were randomly selected from the 85 patients and these 24 image sets were evaluated twice. Consequently, 279 image sets were analyzed by each radiologist. Image interpretation was performed using both 2.5-mm and 0.625-mm-thick axial images as necessary. In addition to the default preselected window settings [window width (WW), 290 HU, window level (WL), 45 HU], radiologists were allowed to change the WW and WL for ease of assessment. Images were shown in a random manner not in a side-by-side way. Both radiologists were blinded to patient data, clinical information and image reconstruction techniques.

For each image data set, each radiologist graded subjective image noise, artifacts, critical reproduction of visually sharp anatomical structures (namely, the ureter) and diagnostic acceptability. Image quality characteristics assessed in this study have been described in the European Guidelines on Quality Criteria for Computerized Tomography (EUR16262 2011) and have been used in multiple previous studies in the radiology literature (Prakash et al. 2010; Singh et al. 2010; Desai et al. 2012). Subjective image noise was assessed by a five-point scale (1 = minimal image noise, 2 = less than average noise, 3 = average image noise, 4 = above average noise, and 5 = unacceptable image noise). Streak artifact (at the level of sacroiliac joint level) and blotchy pixelated appearance (in liver parenchyma) were assessed by a three-point scale (1 = artifacts unapparent or only minimally recognizable, 2 = artifacts recognized but not interfering with diagnostic decision making, and 3 = substantial artifacts recognized affecting diagnostic decision making). Depiction of ureter was assessed by a four-point scale (1 = visible for all length, 2 = not visible in pelvis, 3 = not visible in pelvis and somewhere else, 4 = not visible at all). Diagnostic acceptability was assessed by a four-point scale (1 = fully acceptable, 2 = probably acceptable, 3 = deemed acceptable only for limited clinical conditions, and 4 = unacceptable).

The radiologists were also involved in a lesion detection study for adrenal nodule. Each lesion was evaluated for their location and certainty level (0 = no lesion, 1 = lesion probably not present, 2 = lesion presence equivocal, 3 = lesion probably present, and 4 = lesion definitely present), according to the free-response receiver operating characteristics (FROC) paradigm.

A consensus panel of two different radiologists (M.K. and K.Y. with 4 and 2 years of experience, respectively) independently interpreted the entire set of images from R-FBP, and identified the lesions. Interpretation of images for establishment of the reference standard occurred both as a free search through the CT sections and as a directed analysis of all candidate lesions originally identified by the previous two radiologists (J.S. and I.M.). The panel members (M.K. and K.Y.), without knowledge of the source of detection of the candidate lesions, assessed each candidate and arrived at a final consensus decision as to whether the finding represented a lesion (true-positive finding) or not (false-positive finding). CT attenuation and longest diameter of adrenal nodules were measured by one of the panel members (K.Y.), by placing a circular ROI centrally over the two thirds of the nodule.

### Statistics

The data were analyzed using JMP 9.0.0 software (SAS Institute, Cary, NC, USA). Objective image data and CT attenuation of adrenal nodules between each reconstruction algorithm were compared using the Student’s paired t-test. Subjective image data was compared using the sign test. To analyze interobserver and intraobserver agreement, Cohen’s weighted kappa analysis was performed. The following kappa values were used to indicate agreement; 0 – 0.20 (poor agreement), 0.21 – 0.40 (fair agreement), 0.41 – 0.60 (moderate agreement), 0.61 – 0.80 (good agreement), and 0.81 – 1.00 (excellent agreement). Sensitivity of adrenal nodule certainty was calculated (certainty levels of 4 and 3 were defined as positive, those of 2 to 0 as negative). The sensitivity data were analyzed using McNemar’s test. For the results including diagnostic certainty levels of adrenal nodule, jackknife alternative free-responsereceiver-operating characteristic (JAFROC) analysis was performed. JAFROC analysis has been proposed for estimating the statistical significance of differences between modalities when location issues are relevant, and has been widely used in multiple previous studies in the radiology literature (Yamada et al. 2012; Yanagawa et al. 2009; Hirose et al. 2008). JAFROC analysis is based on a free-response receiver operating characteristic (FROC) paradigm and accounts for reader variation. Conventional ROC analysis is of limited value for this kind of application, because only one response per image can be used per case, and the location of the response cannot be taken into account in the evaluation. In contrast, FROC analysis allows evaluation of the performance of radiologists in diagnosing medical images using multiple responses, each with information on the diagnostic certainty level and location. For statistical testing of differences in radiologist performance between L-ASIR, UL-ASIR, and UL-MBIR, JAFROC version 4.0 software (http://www.devchakraborty.com) was applied to estimate figure-of-merit (FOM) values (analog of the area under the ROC curve, defined as the probability that a true-positive confidence rating exceeds any false-positive rating on cases without lesions) of each modality (L-ASIR, UL-ASIR, and UL-MBIR) with 95% confidence intervals. An F-test was used internally for the analysis of variance, yielding a P value for rejecting the null hypothesis of no difference between the three modalities. For comparing multiple groups, the Bonferroni correction was applied and P<0.016 was considered to suggest statistically significant difference.

## Results

### Radiation dose

Radiation dose descriptors for abdominopelvic CT examinations acquired with RDCT, LDCT, and ULDCT for all 85 patients are summarized in Table 1. Compared with LDCT, there was 63% reduction of DLP with ULDCT.

**Table 1:** **Radiation doses in each CT scan**

	Referential-dose	Low-dose	Ultralow-dose
CTDIvol (mGy)	7.99± 2.90	1.89± 0.58	0.70± 0.22
DLP (mGy cm)	410 ± 166	97± 34	36 ± 13
ED (mSv)	6.15 ± 2.49	1.45 ± 0.50	0.54 ± 0.19

### Objective image analysis

The results of objective image analysis are summarized in Table 2. UL-MBIR had significantly lower image noise than L-ASIR and UL-ASIR (p<0.01 between all pairs for both abdominal aorta and iliopsoas muscle). There were no significant differences in CT number of the iliopsoas muscle between UL-MBIR, L-ASIR and UL-ASIR (p=0.10–0.44), while CT numbers of the abdominal aorta of L-ASIR and UL-ASIR were significantly higher than that of UL-MBIR (p<0.01 for both).

**Table 2:** **Comparison of objective image data**

		Image noise or CT number (HU)			Comparison (P value)		
		UL-MBIR	L-ASIR	UL-ASIR	UL-MBIR vs. L-ASIR	UL-MBIR vs. UL-ASIR	L-ASIR vs.UL-ASIR
Image noise	Aorta	18.1 ± 3.5	47.4 ± 6.6	90.5 ± 11.1	<0.001*	<0.001*	<0.001*
	Muscle	17.2 ± 2.7	43.9 ± 6.2	80.8 ± 9.1	<0.001*	<0.001*	<0.001*
CT number	Aorta	39.8 ± 5.7	43.6 ± 6.3	45.4 ± 9.5	<0.001*	<0.001*	0.085
	Muscle	58.5 ± 9.2	57.6 ± 8.8	59.1 ± 10.5	0.125	0.441	0.096

For comparison, Student’s paired t-test was performed.

*P<0.016.

### Subjective image analysis

Interobserver agreements of subjective image evaluation were fair to good, except for streak artifact (poor). Intraobserver agreements for reader 1 were fair to excellent, except for streak artifact (poor) and diagnostic acceptability (poor). Those for reader 2 were moderate to excellent, except for streak artifact (fair).

**Figure 1:**
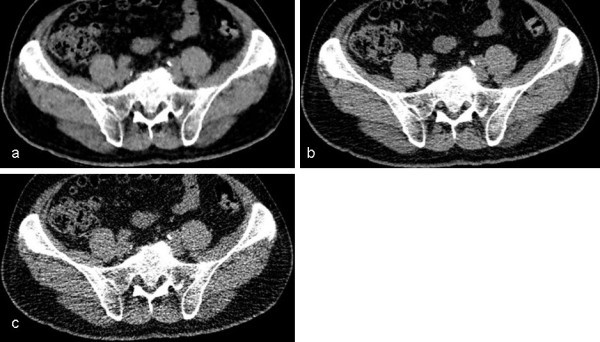
**Noise and streak artifact of each CT image.** Axial unenhanced CT images of UL-MBIR (**a**), L-ASIR (**b**), and UL-ASIR (**c**) of a 61-year-old man weighing 75 kg. Less noise and fewer streak artifacts are seen in UL-MBIR than in L-ASIR and UL-ASIR.

**Figure 2:**
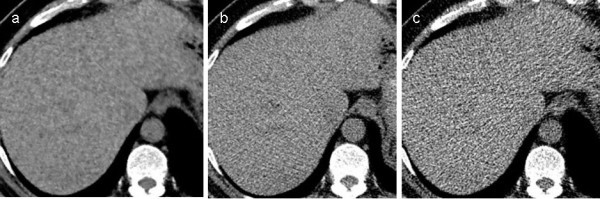
**Blotchy pixelated appearance of each CT image.** Axial unenhanced CT images of UL-MBIR (**a**), L-ASIR (**b**), and UL-ASIR (**c**) of a 61-year-old man weighing 75 kg, the same patient as in Figure 1. A blotchy pixelated appearance is uniquely seen in UL-MBIR images.

The results of subjective image analysis are summarized in Table 3. UL-MBIR was significantly better than L-ASIR and UL-ASIR for subjective image noise (p<0.01 between all pairs for both readers) and streak artifacts (p<0.01 between all pairs for both readers) (Figure 1). UL-MBIR was more often associated with pixelated blotchy appearance compared with ASIR images (p<0.01 for both readers) (Figure 2). UL-MBIR was significantly better for depiction of ureters than UL-ASIR (p<0.01 for both readers), while there was no significant difference when compared to L-ASIR (p>0.21 for both readers). UL-MBIR was also significantly better for diagnostic acceptability than UL-ASIR (p<0.01 for both readers), while there was no significant difference compared with L-ASIR (p>0.65).

**Table 3:** **Comparison of subjective image data**

		Score			Comparison (P value)		
		UL-MBIR	L-ASIR	UL-ASIR	UL-MBIR vs. L-ASIR	UL-MBIR vs. UL-ASIR	L-ASIR vs. UL-ASIR
Noise (1/2/3/4/5)	Reader 1	0/1/81/3/0	0/0/64/21/0	0/0/3/81/1	<0.001*	<0.001*	<0.001*
	Reader 2	1/5/55/24/0	0/0/7/76/2	0/0/1/10/74	<0.001*	<0.001*	<0.001*
Streak artifact (1/2/3)	Reader 1	73/12/0	40/45/0	25/56/4	<0.001*	<0.001*	0.014*
	Reader 2	24/60/1	1/74/10	1/4/80	<0.001*	<0.001*	<0.001*
Pixelated blotchy appearance (1/2/3)	Reader 1	4/74/7	84/1/0	83/1/1	<0.001*	<0.001*	1.000
	Reader 2	0/19/66	83/1/1	84/1/0	<0.001*	<0.001*	1.000
Depiction of ureter (1/2/3/4)	Reader 1	15/41/26/3	10/48/25/2	3/32/43/7	1.000	<0.001*	<0.001*
	Reader 2	39/25/13/8	42/21/16/6	14/29/34/8	0.210	<0.001*	<0.001*
Diagnostic acceptability (1/2/3/4)	Reader 1	0/11/73/1	0/14/70/1	0/0/57/28	0.648	<0.001*	<0.001*
	Reader 2	29/30/19/7	31/30/14/10	1/2/19/63	0.775	<0.001*	<0.001*

For comparison, sign test was performed.

*p<0.016.

**Figure 3:**
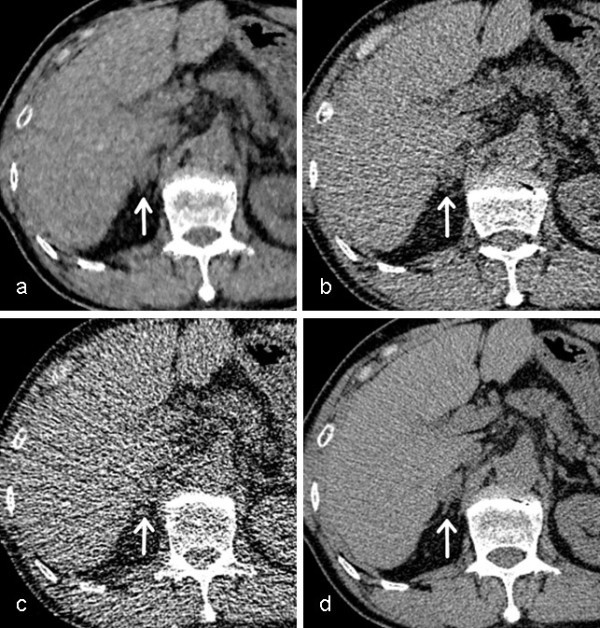
**The adrenal nodule in each CT image.** An adrenal nodule with 20 mm in longest diameter (white arrow) in axial unenhanced CT images of UL-MBIR (**a**), L-ASIR (**b**), UL-ASIR (**c**) and R-FBP (**d**) of a 65-year-old man weighing 61 kg. In UL-ASIR (**c**), the margin of this adrenal nodule is obscure due to the prominent image noise. The certainty level (UL-MBIR/L-ASIR/UL-ASIR) for this lesion by each reader was 4 (lesion definitely present)/3(lesion probably present)/3 by reader 1 and 3/3/0(no lesion) by reader 2, respectively.

The consensus panel identified 12 adrenal nodule positive patients (Figure 3). The total number of adrenal nodules was 15. Mean longest diameter of adrenal nodules was 20.9 ± 10.2 mm (range; 11.6 – 50.9 mm). CT attenuation of adrenal nodules in UL-ASIR (27.8 ± 24.2 HU) was significantly higher than in UL-MBIR (18.6 ± 20.0 HU) (p<0.01). No significant differences were seen between UL-MBIR and L-ASIR (22.5 ± 19.3 HU) (p=0.04), or between L-ASIR and UL-ASIR (p=0.14).

Sensitivity data for detection of adrenal nodules is shown in Table 4. L-ASIR was significantly better than UL-ASIR for one radiologist (p=0.01), however no significant differences were identified among remaining pairs for both radiologists (Sensitivity = 0.27-0.67, p>0.08). FOM values for adrenal nodules are shown in Table 5. No significant differences were identified among all pairs (FOM = 0.61-0.86, p>0.12).

**Table 4:** **Sensitivity for lesion detection study**

	Sensitivity			Comparison (p value for sensitivity)		
	UL-MBIR	L-ASIR	UL-ASIR	UL-MBIR vs. L-ASIR	UL-MBIR vs. UL-ASIR	L-ASIR vs. UL-ASIR
Radiologist 1	0.67	0.67	0.47	1.00	0.18	0.08
Radiologist 2	0.53	0.67	0.27	0.32	0.10	0.01*

For comparison, McNemar’s test was performed.

*p<0.016.

**Table 5:** **Figure of merit values for lesion detection study**

	Figure of merit			Comparison (p value)		
	UL-MBIR	L-ASIR	UL-ASIR	UL-MBIR vs. L-ASIR	UL-MBIR vs. UL-ASIR	L-ASIR vs. UL-ASIR
Radiologist 1	0.86	0.86	0.80	1.00	0.54	0.54
Radiologist 2	0.77	0.79	0.61	0.87	0.17	0.12

JAFROC analysis was performed to compare the figure of merit of adrenal nodule.

No significant difference was seen between reconstruction algorithm.

## Discussion

In this prospective study of 85 patients, the effect of MBIR on radiation dose reduction was evaluated and image quality and diagnostic performance was compared with ASIR. In abdominopelvic CT images acquired with nearly 63% less radiation (ULDCT), significant improvements in image noise and streak artifacts were observed with the use of MBIR compared with ASIR. No significant differences were seen between UL-MBIR and L-ASIR with regards to diagnostic acceptability. To the best of our knowledge, this is the first prospective clinical study in the abdominopelvic region to evaluate CT radiation dose reduction in the same patients and compare image quality characteristics with two different reconstruction methods: MBIR and ASIR.

The present results of MBIR that show significant improvement in image noise, streak artifacts, and diagnostic acceptability over ASIR in dose-reduced abdominopelvic CT are consistent with the results from previous reports that evaluated dose reduction and image quality characteristics of MBIR in chest CT (Yamada et al. 2012; Katsura et al. 2012). Recently, Singh et al. evaluated MBIR, ASIR, and FBP images from 10 patients by comparing them in a side-by-side manner and reported that MBIR renders acceptable image quality and diagnostic confidence in 50 mAs abdominal CT images while FBP and ASIR do not (Singh et al. 2012). The present study differs from their study in that we prospectively and intraindividually evaluated 85 patients by performing image analysis in a blinded and randomized manner. The present results indicate the substantial potential of MBIR for achieving further radiation dose reductions over ASIR without severely compromising image quality.

The CT number in the abdominal aorta was significantly higher in L-ASIR and UL-ASIR than in UL-MBIR. The CT number of the adrenal nodules was also significantly higher in UL-ASIR than in UL-MBIR. This may be due to the substantial streak artifacts seen in ASIR images, which could increase CT number, according to Yamada et al. (2012).

Interobserver and intraobserver agreement concerning the streak artifact were poor or fair. This may be due to the difficulty in distinguishing streak artifact from image noise, particularly when there is prominent image noise. Intraobserver agreement for diagnostic acceptability was poor for one of the readers. Since diagnostic acceptability is a more notional evaluation term, evaluation basis could have changed during evaluation of 279 images, and reflect the reader becoming accustomed to the images.

Unique image features noted in the MBIR-reconstructed images in the present study include a pixelated blotchy appearance. The exact reasons for this MBIR-unique appearance remain unknown, and may be due to inherent differences in image reconstruction. A pixelated blotchy appearance has been described in many initial ASIR reports (Prakash et al. 2010; Singh et al. 2010). However, this appearance was not prominently seen on ASIR images in the present study, which, according to the vendor, can be attributed to the advancements of the ASIR algorithm that have been made following the earlier studies. In the present study, the difference in image appearance and lack of reader familiarity may have contributed to the variability between the readers, particularly in the subjective evaluation of some image artifacts and anatomical structures. This variability should decrease over the course of familiarization with MBIR images and may decrease even more if the MBIR-unique image appearance becomes less prominent with further advancement of the MBIR algorithm. Overall, these MBIR-unique image features were not overly distracting in the present study, and had little effect on diagnostic acceptability.

Though sensitivity for detection of adrenal nodules of L-ASIR was significantly better than UL-ASIR in reads from one radiologist, the remainder of the lesion detection study did not show significant differences between MBIR and ASIR. This may be due to the low prevalence of lesions. Since our results of image quality assessment strongly support the potential of MBIR for dose-reduced CT in certain clinical situations, future studies with an increased number of patients (the sample size should be determined by statistical power analysis) specifically aimed at assessing lesion detectability are necessary.

Several limitations of this study must be considered. First, the body size of the patients was generally small. MBIR has not yet been assessed in extremely large or obese patients, and this needs to be investigated in future studies. Second, although MBIR is expected to be helpful for dose reduction in pediatric patients, they were not included in the present study. Third, all of the abdominopelvic CT examinations were performed without intravenous contrast medium administration. Future studies should include assessment of contrast enhancement in MBIR-reconstructed images. Fourth, the results may not apply to other similar iterative reconstruction methods available from other vendors. Fifth, owing to the difference in image appearance, blinding of the radiologists between MBIR and ASIR during subjective image analysis was difficult. However, the image sets acquired with different dose levels and reconstruction techniques were randomized. Finally, whether UL-MBIR is ready for clinical use was not investigated thoroughly, because of the low number of adrenal lesions. And the sensitivity for adrenal detection in UL-MBIR was not sufficiently high, therefore its use in clinical setting might be limiting. However, from our study and some previous studies (Mieville et al. 2013; Singh et al. 2012; Katsura et al. 2012), it has become evident that MBIR can achieve radiation dose reduction without severely compromising image quality compared to ASIR, future studies on this issue especially for those susceptible to x-ray radiation such as children, adolescents, and immunocompromised patients, are expected.

In conclusion, MBIR significantly improves image noise and streak artifacts compared to ASIR in abdominopelvic CT. MBIR shows greater potential than ASIR for achieving further radiation dose reductions over ASIR without severely compromising image quality.
